# Retrieval-enhanced drafting of ClinicalTrials.gov data elements from clinical protocols

**DOI:** 10.1017/cts.2026.10735

**Published:** 2026-03-31

**Authors:** Ramya Sri Baluguri, Nicholas Anderson

**Affiliations:** University of California Davis, USA

**Keywords:** Clinical trial protocols, natural language processing, data extraction, ClinicalTrials.gov, artificial intelligence

## Abstract

**Background:**

Manual submission of clinical trial data to the ClinicalTrials.gov registry is labor-intensive and error-prone, contributing to variability in the completeness and consistency of registry entries. To explore whether recent advances in large language models could support this process, we developed ChatCT, a pilot retrieval-augmented system that drafts ClinicalTrials.gov registry elements.

**Methods:**

We evaluated ChatCT-generated registry elements across three dimensions: 1. semantic similarity to the public ClinicalTrials.gov record, 2. formatting compliance with ClinicalTrials.gov requirements, and 3. coverage of key trial biomedical concepts.

**Results:**

ChatCT-generated registry elements were highly semantically similar to human-authored ClinicalTrials.gov records (median BERTScore F1 ≈ 0.82). Formatting compliance was high for structured elements, including Study Design (91% of required fields present; mean completeness 0.897) and Arms/Interventions (75%; 0.772), while narrative sections showed greater variability, including Outcome Measures (79%; 0.929) and Study Description (57%; 0.784). Ontology-based concept extraction and matching demonstrated consistently high precision, with scores ranging from 90% to 100%.

**Conclusions:**

A retrieval-augmented large language model can generate ClinicalTrials.gov registry drafts that preserve essential protocol details and adhere to most formatting requirements. However, light post-processing (e.g., automated schema validation) remains necessary for full submission readiness. This proof-of-concept evaluation suggests that ChatCT-assisted drafting could support registry reporting by improving consistency between protocol documents and publicly reported trial information.

## Introduction

Clinical trial registries such as ClinicalTrials.gov were established to improve transparency and accountability in research by ensuring that key information about trial protocols and results is publicly available [1]. Registration and results reporting on ClinicalTrials.gov is required for studies that meet the definition of Applicable Clinical Trials (ACTs) under the FDA Amendments Act of 2007. ACTs generally include most interventional studies of FDA-regulated drugs, biologics, or devices conducted in the United States or under FDA jurisdiction, with notable exclusions such as Phase 1 drug trials and small feasibility device studies [2]. NIH policy is broader than the statute, requiring all NIH-funded clinical trials – including early-phase and non-FDA-regulated interventions – to register and report on ClinicalTrials.gov regardless of ACT status [3]. Despite these published requirements, timely and accurate compliance remains challenging. Between 2019 and 2022, up to 16–18% of NIH-funded trials were registered late, and only about half had their results posted within mandated timeframes [4]. These gaps highlight the ongoing operational burden of translating complex protocols into ClinicalTrials.gov’s structured fields and processes, which are prone to delays, omissions, and inconsistencies across institutions [5].

Responsible parties – including individual investigators, study teams, and institutional clinical trial support staff – must restate protocol elements – study design, eligibility, interventions, and outcomes – and study-specific operational details (e.g., timelines, safety monitoring) in the defined formats required by ClinicalTrials.gov [6–8]. Beyond timeliness, the completeness and quality of registry entries are inconsistent. Many registry records are incomplete or out-of-date, lacking important information or updates [9]. Free-text outcome descriptions and eligibility criteria are sometimes underspecified or inconsistent, which can make it difficult to interpret or compare trials [10]. Notably, comparisons of registry entries to trial protocols and publications have documented cases of discrepancies and changes in outcomes or other details that were not transparently reported [11]. Moreover, inconsistencies occur alongside real-world challenges. Individual studies often have protocol documentation reflecting complex, multi-year histories, and complete and timely registration within a single organization can be influenced by a range of sociotechnical factors, including staff or investigator turnover, limited availability of relevant expertise, lack of familiarity with study nomenclature, and changes in registration processes – all of which increase the risk of delayed, incomplete, or inconsistent registry entries.

## Related work

The use of artificial intelligence (AI) methods to assist clinical documentation is gaining traction. Early evaluations suggest that retrieval-augmented large language models (LLMs) outperform base models on domain-specific tasks by grounding their outputs in source documents [12,13]. This is consistent with broader reports that Retrieval-Augmented Generation (RAG) pipelines can match or exceed expert performance while also reducing unsupported content; for example, a study reported that a GPT-4 RAG workflow achieved 96.4% accuracy versus 86.6% for physicians on a structured preoperative assessment, with substantially reduced hallucinations [14].

Clinical trial protocols contain many of the core elements required for structured trial reporting (e.g., population, interventions, outcomes, timelines) but are typically presented in narrative formats that must be translated into a rigid schema referencing controlled vocabularies necessary for registries and downstream use [15]. AI and natural language processing (NLP) methods have been widely studied for extracting structured trial content – most notably eligibility criteria parsing and representation – from ClinicalTrials.gov-linked protocol text and trial records [16]. Recent reviews further describe growing use of LLMs to enhance clinical trial workflows, predominantly focusing on information extraction and trial data management tasks [17]. However, applying these methods on ClinicalTrials.gov Protocol Registration and Results System (PRS) requires not only correct content but also adherence to specific syntactic field structures, definitions, and review criteria, creating a compliance-focused documentation problem distinct from general information extraction [18].

Rationale: Given the regulatory requirements for ClinicalTrials.gov reporting and the limitations of existing approaches to structuring protocol-derived information, there is a need to better understand how recent advances in retrieval-augmented large language models might be applied in this context.

## Objective

In this study, we present ChatCT, a prototype retrieval-augmented GPT-4–based system for drafting ClinicalTrials.gov registry entries from Institutional Review Board (IRB)-approved clinical trial protocols. The objective of this pilot evaluation was to assess the feasibility, accuracy, and quality of ChatCT’s performance using a held-out set of retrospective, completed trial protocols paired with their corresponding ClinicalTrials.gov reference registrations. Evaluation was conducted across three dimensions: 1. semantic fidelity of ChatCT-generated content relative to human-authored registry entries, 2. adherence to ClinicalTrials.gov PRS syntactic and structural requirements, and 3. protocol-grounded clinical concept coverage.

## Materials and methods

We conducted a single-institution, retrospective evaluation of the ChatCT system using IRB-approved protocols managed through the UC Davis IRB office and paired with their extracted public ClinicalTrials.gov entries. The study was a pragmatic, descriptive analysis focusing on performance characterization rather than formal hypothesis testing. We identified a cohort of clinical trials at our academic medical center that had both an IRB-approved protocol document and a corresponding registration on ClinicalTrials.gov under our organization’s account. Starting from an IRB database export (approximately 1,072 studies), we applied the following inclusion criteria: 1. Trials initiated at UC Davis with an IRB-approved protocol available in the institutional system; 2. Matching ClinicalTrials.gov record in our PRS account (confirmed via IRB identifier cross-check); and 3. Study status marked closed or terminated (to ensure a final version of the protocol and a completed registry entry were available). We excluded protocols that were not expected to be registered by our institution (e.g., multi-site trials in which another sponsor was responsible, studies not meeting the ACT criteria or exempt from registration, sub-studies filed under a parent National Clinical Trial (NCT) number, and administrative closures). For each included trial, we retrieved the latest IRB-approved protocol and the publicly available ClinicalTrials.gov record. This process yielded a set of 15 protocols and matched registry entries for system testing, and 29 protocols and matched registry entries for system evaluation.

## System overview

### ChatCT architecture

**Figure 1.**
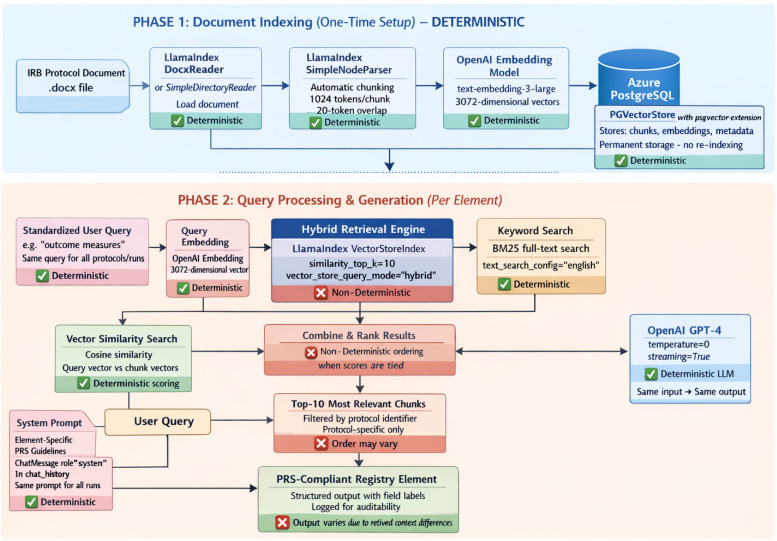
Architecture and data flow: Phase 1 illustrates the one-time, deterministic document ingestion, chunking, embedding, and storage to a pgvector-enabled PostgreSQL database. Phase 2 depicts per-element query processing, hybrid retrieval (vector similarity and keyword search), prompt assembly, and generation using a temperature-zero LLM. Components are labeled to distinguish deterministic steps from non-deterministic retrieval stages, which may affect retrieved context ordering and downstream outputs.

The system utilizes a RAG pipeline comprising two main phases: 1. Document indexing and retrieval of relevant protocol text, and 2. Query processing and generation of structured registry elements (Figure 1). In the ChatCT retrieval step, protocols were loaded via LlamaIndex and chunked using a default text splitter (≈1024 tokens per chunk with a 20-token overlap). The fixed token overlap preserved local continuity across chunk boundaries, and retrieving multiple chunks reduced the chance of missing key trial details (e.g., eligibility criteria spanning multiple pages). The indexed chunks are stored in a PGVector and use a hybrid search to retrieve the top-k (*k* = 10) most relevant chunks per query. Hybrid retrieval combines vector similarity search (cosine similarity) and keyword search and may return chunks in different orders across runs (e.g., user asking for outcomes element of same protocol for 2 or more times) when similarity scores are tied or very close – a known characteristic of hybrid retrieval systems that reflects inherent non-determinism in ranking when multiple chunks have equivalent relevance scores. When the user requests a specific registry element (for example, “Study Design” or “Outcome Measures”), ChatCT performs a semantic similarity search on the indexed protocol to fetch the most relevant passage(s) related to that element. Importantly, the retrieval is initiated using standardized user queries (using the element name, e.g., “outcome measures” for Outcome Measures) consistently across all protocols and all runs to ensure that the same semantic search strategy is applied. Moreover, the retrieval is restricted to the protocol in question – the model has no access to external data – ensuring that any generated content is grounded in the trial’s actual IRB-approved protocol.

In the constrained generation step, ChatCT produces the textual draft of each registry element. The model is provided with: (a) the retrieved protocol excerpts as context, and (b) a fixed field-specific system prompt template reflecting ClinicalTrials.gov formatting rules. These prompts instruct the model on the expected structure and terminology for that element. For example, when generating the “Arms and Interventions” element, the prompt directs the model: *“List each study arm as a bullet with an Arm Title (Arm Label and Arm Type) followed by the assigned Interventions for that arm.”* Similarly, the Outcome Measures prompt specifies that each outcome should include an Outcome Title, a Description, and a Time Frame. All model inputs and outputs were logged for auditability.

## System validation

To ensure that variation in ChatCT-generated outputs reflected non-deterministic retrieval functions, we froze all system prompts, model parameters, and retrieval settings prior to evaluation. We evaluated 219 ChatCT-generated elements across 29 trials. This consisted of 46 Study Design elements, 47 Arms and Interventions, 52 Outcome Measures, and 74 Study Descriptions. Some protocol–element pairs were generated more than once under the frozen configuration (Appendix 2, Table 1) and were not prespecified. Where run counts vary across protocol–element pairs, we aggregated metrics at the protocol–element level, ensuring each pair contributed a single value to summary statistics.

## Evaluation framework

We performed a structured comparison of ChatCT-generated registry elements (the “candidate”) against two sources: the corresponding ClinicalTrials.gov entry (the human-authored “reference”) and the IRB protocol text (the “source” of truth). The evaluation was structured to process the three key dimensions: 1. pairwise comparison of candidate text and reference for semantic fidelity of candidate, 2. comparing the candidate against ClinicalTrials.gov PRS requirements for syntactic/formatting assessment, and 3. comparing concepts extracted from candidate and reference texts, grounded in the protocol, to examine conceptual consistency.

### Semantic fidelity

We treated the text of the ClinicalTrials.gov entry as the reference standard for content (acknowledging that it is human-curated). We computed BERTScore (using the recommended RoBERTa-Large model) – a transformer-based text similarity metric – to quantify semantic similarity between each candidate and the corresponding reference element [19]. BERTScore aligns tokens in the candidate and reference based on contextual embeddings, thereby measuring semantic overlap [20].

After computing semantic similarity metrics for each candidate–reference pair, we grouped results by unique protocol ID and registry element type (Study Design, Arms and Interventions, Outcome Measures, and Study Description). Within each protocol–element pair, we averaged scores across all candidates generated for that pair. We then computed the overall mean BERTScore precision, recall, and F1 for each element type by averaging these protocol-level means across the 29 protocols. This aggregation strategy ensured that each protocol contributed equally to the overall semantic similarity metrics, regardless of the number of candidates generated per pair.

### Syntactic/formatting compliance

Each registry element is governed by data element definitions and formatting requirements specified by ClinicalTrials.gov (Table 1). We used these definitions to develop a post hoc, automated rule-based checklist (Appendix 1, Table 1B) that evaluated each ChatCT-generated candidate for the presence and correctness of required structural subfields. All required subfields for each registry element were assessed individually.

**Table 1.** Error categories, penalty weights, and illustrative examples used to evaluate PRS formatting compliance

Error Category	Penalty	Definition	Example
Missing	−0.30	The required PRS field is completely absent.	Primary Purpose: Treatment; Study Phase: Phase 2 (but Study Type not present)
Invalid Value	−0.30	Field value does not conform to PRS-controlled vocabulary.	Study Phase: Phase II (invalid; PRS requires Phase 2)
Length Violation	−0.30	Field content exceeds PRS character limits.	Intervention Description exceeds 1,000 characters
Vague Time Frame	−0.20	Time Frame provided, but non-specific or ambiguous.	Time Frame: up to one year
Missing Component	−0.10	Structured composite field present but missing one or more required components.	Outcome Measure includes Title and Description but lacks Time Frame
Format Issue	−0.10	Field present but improperly formatted, unlabeled, or merged with other content.	“The study uses a double-blind design…” (Masking information present but unlabeled)

PRS = Protocol Registration and Results System.

For example, an interventional *Study Design* candidate must explicitly include the fields *Allocation, Intervention Model, Masking, Phase,* and *Primary Purpose* and their labels using PRS-approved terminology, while the *Arms and Interventions* element must list each study arm separately with an Arm Label and Arm Type preceding the intervention description. We encoded these requirements programmatically and evaluated each candidate element for both the presence and proper formatting of each required subfield (see Table 1A; full scoring rubric provided in Appendix 1, Table 1B).

Scoring began at 1.0 for each element, and penalties were applied according to predefined error categories aligned with PRS review criteria in Table 1. We did not implement synonym or vocabulary normalization beyond basic case-insensitive matching; therefore, candidate text was required to contain the expected fields, labels, and structure to receive full credit. For instance, if PRS expects “Masking: Double” and the ChatCT output says “double-blind” without using the exact allowed term, it would not receive full credit for that field.

For the *Outcome Measures* element, which comprises multiple components per outcome, each outcome was evaluated for inclusion of an Outcome Title, Description, and Time Frame. Partial credit was assigned when one or more components were present, and penalties were applied (+0.1 per component found, −0.2 for invalid *Time Frame* formats) for missing components or invalid or vague Time Frame formats (e.g., non-specific durations). Outcomes missing one or more required components were categorized as a *missing component* error type.

From these subfield-level checks, we then computed two summary metrics for each candidate: 1. an overall formatting score, defined as the mean of subfield scores on a 0–1 scale (where 1.0 indicates all required subfields were present and correctly formatted), and 2. a compliance rate, defined as the percentage of required subfields scoring ≥0.70, which we interpreted as “passing.”

These metrics reflect how close the ChatCT output was to a submission-ready format, independent of content accuracy.

### Clinical concept coverage and consistency

To assess whether ChatCT captured accurate biomedical concepts for each generated registry draft, we performed an ontology-based concept analysis. Biomedical concepts were extracted using the SciSpaCy NLP library with a biomedical named entity recognition (NER) model and a UMLS biomedical entity linker [21,22] from: 1. source chunks, 2. reference, and 3. candidate texts. These extracted entities were normalized to standard identifiers across four vocabularies: UMLS Concept Unique Identifiers (CUIs) for general biomedical terms, SNOMED CT codes for clinical concepts, MeSH terms for conditions, and RxNorm codes for interventions [23].

Following concept extraction, we implemented an automated ontology-based matching layer to evaluate concept grounding and coverage. For ChatCT performance evaluation at the concept-coverage level, each ChatCT-generated output was treated as an individual data point. Concepts extracted from each candidate were matched against the corresponding protocol source chunks retrieved during that generation, reflecting the non-deterministic nature of RAG. Concepts extracted across all ChatCT outputs were aggregated to characterize overall concept coverage. Concepts that were not automatically matched by the ontology layer to protocol source chunk–derived concepts were flagged for manual review. Manual verification was performed only for ChatCT-generated outputs to determine whether unmatched concepts reflected unsupported content or limitations of the automated matching layer. After assessing concept coverage, we evaluated concept consistency by comparing ChatCT-generated concepts, reference concepts, and protocol source chunk–derived concepts. Because ClinicalTrials.gov entries represent a single finalized human-authored record per protocol–element pair, whereas ChatCT outputs may be generated one or more times per protocol–element and depend on retrieval-augmented context, distinct concept counting strategies were applied. To avoid inflation from repeated generations, ChatCT-generated concepts were unioned across multiple candidates within each protocol–element before computing concept-level precision, recall, and F1, ensuring each unique concept was counted at most once per protocol–element and evaluated against concepts aggregated from the corresponding protocol source chunks. In contrast, concepts extracted from ClinicalTrials.gov reference entries were counted once per protocol–element and evaluated against the same protocol source chunk–derived concept set.

## Results

We present the results following the logical order of a ClinicalTrials.gov submission, addressing in turn: 1. The semantic fidelity of the ChatCT-generated content to their corresponding ClinicalTrials.gov reference entries, 2. the formatting compliance of the ChatCT drafts with ClinicalTrials.gov PRS requirements, and 3. the clinical concept coverage.

## Semantic alignment with ClinicalTrials.gov reference entries

**Figure 2.**
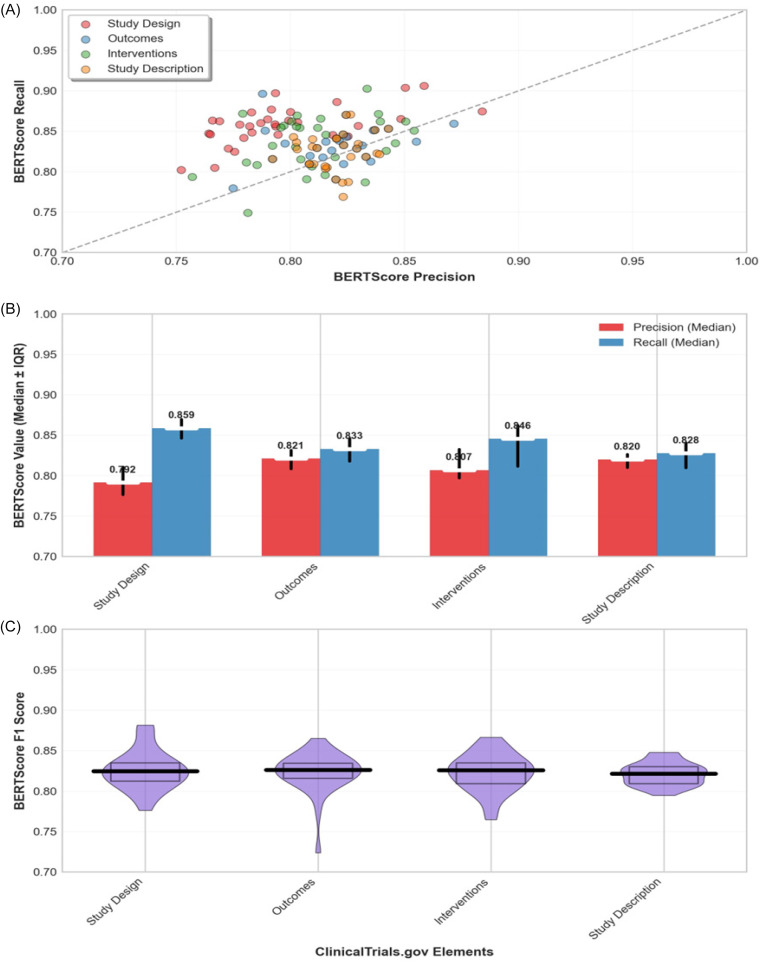
Semantic alignment of ChatCT-generated content with human entries. A. Precision and recall for each generated element, B. Median precision (blue) and recall (orange) achieved for each element, and C. Distribution of BERTScore F_1_ scores for ChatCT outputs vs. reference text in each element.

Across all studies and registry elements, content generated by ChatCT was highly similar to the corresponding ClinicalTrials.gov entries. The mean BERTScore F1 between ChatCT-generated and reference text was 0.82. As shown in Figure 2, performance was consistently high across all four registry elements. Figures 2A and 2B show that ChatCT drafts tended to exhibit slightly higher recall than precision when compared with human-authored entries, indicating that the generated content generally captured most information present in the reference text (high recall) while occasionally including additional details (slightly lower precision).

In practice, this pattern reflects the model’s ability to faithfully reproduce human-entered content while incorporating relevant details from the source IRB protocol that were not explicitly included in the registry entry. For example, a ChatCT-generated *Outcome Measures* element might include a secondary endpoint specified in the protocol that was omitted from the corresponding ClinicalTrials.gov record.

Median BERTScore F1 values were 0.81 for *Study Design*, 0.82 for *Arms and Interventions*, 0.82 for *Outcome Measures*, and 0.82 for *Study Description*, with relatively narrow interquartile ranges (approximately ±0.015–0.020 for each element). Figure 2C illustrates the distribution of BERTScore F1 scores across registry elements.

## Syntactic and formatting compliance

**Figure 3.**
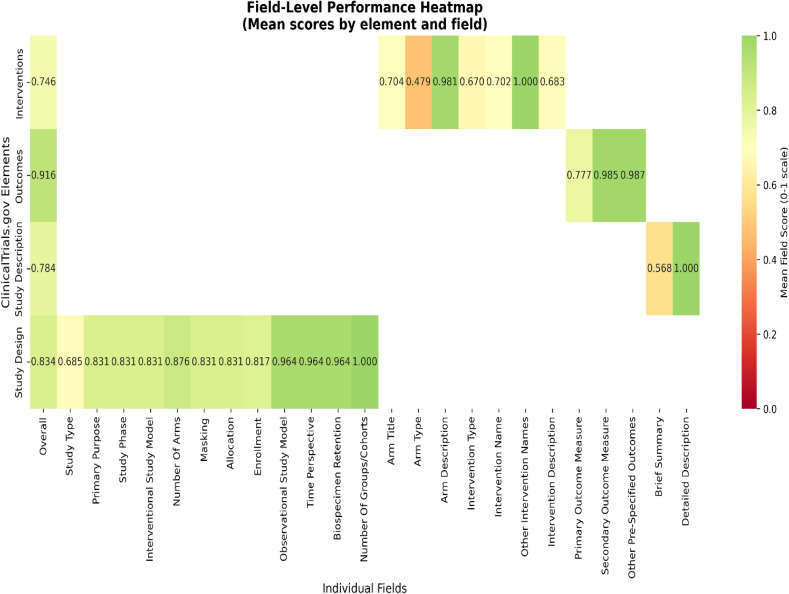
Rows represents one of the four ClinicalTrials.gov elements generated by ChatCT, each column corresponds to a specific required field within that element (e.g., allocation, masking, study phase, etc., for study design).

The *Study Design* mean overall formatting score was 0.897 and achieved the highest structural compliance rate: 91.3% of the 46 Study design outputs contained all required subfields with acceptable formatting. In practice, this means that in the vast majority of candidates, ChatCT successfully listed key design elements such as Allocation, Masking, Phase, etc., using the expected labels and terminology. The few *Study Design* outputs that did not receive full marks usually contained the necessary information in a narrative form but failed to label one or two fields explicitly. For example, a ChatCT-generated *Study Design* paragraph says, “This is a randomized, double-blind Phase 2 trial of …,” which *implies* Allocation, Masking, and Phase, but if it did not break them out as separate labeled lines (e.g., Allocation: Randomized; Masking: Double), it was marked down. In a couple of cases, a minor field was omitted entirely (most often the Phase or Primary Purpose), but we did not encounter any instance in which a major design attribute was completely missing from the content. Arms and Interventions mean formatting score: 0.772 and showed strong but imperfect compliance: 74.5% of 47 outputs met requirements. Common issues included combining multiple arms into a single bullet point rather than maintaining separate entries and omitting Arm Type/Title labels when listing interventions. Outcome Measures mean score: 0.929 and had intermediate compliance: 78.8% of 52 outputs provided all required components. Time Frame validation was the primary failure point, often due to vague descriptions (“up to one year”) rather than specific time points required by ClinicalTrials.gov. Study Description mean score: 0.784 and showed the lowest compliance: only 56.8% of 74 outputs were properly separated Brief Summary and Detailed Description fields. Most failures involved producing single-narrative blocks rather than distinct subsections, which required human editing to split the content appropriately. Overall, the majority of outputs contained relevant information but required formatting adjustments to meet structural requirements. Figure 3 provides a field-level view of syntactic and formatting performance across ClinicalTrials.gov elements, showing mean subfield scores aggregated across all generated candidates. The evaluation system’s strict adherence to ClinicalTrials.gov field definitions – without vocabulary normalization – ensured that only properly formatted, submission-ready content received full compliance scores.

## Clinical concept coverage and consistency

Across all four registry elements, a total of 1368 biomedical concepts were extracted from all ChatCT-generated outputs. Using the automated ontology-based validation layer, 1319 concepts (96.4%) were successfully matched to concepts derived from source chunks at the time of generation, while 49 concepts (3.6%) were flagged as unmatched. Manual review of these unmatched concepts by both authors confirmed that all were supported by the source chunks retrieved at the time of generation. Some unmatched concepts represented direct matches that were not captured by the automated matching process, while others reflected synonymous or semantically equivalent terminology rather than unsupported content. For example, in one case ChatCT produced the broader term “kidney disease,” whereas the retrieved source chunk referenced concepts including “kidney infection,” “kidney damage” and “inflammation of the kidney.” We treated these as specificity mismatches and consider these discrepancies as limitations of our ontology mapping, as contextual semantic alignment was beyond the scope of the manual evaluation process. Systematic handling of synonym-level mappings, specificity mismatch case documentation and contextual semantic alignment will be explored in the projects future evaluation framework.

**Figure 4.**
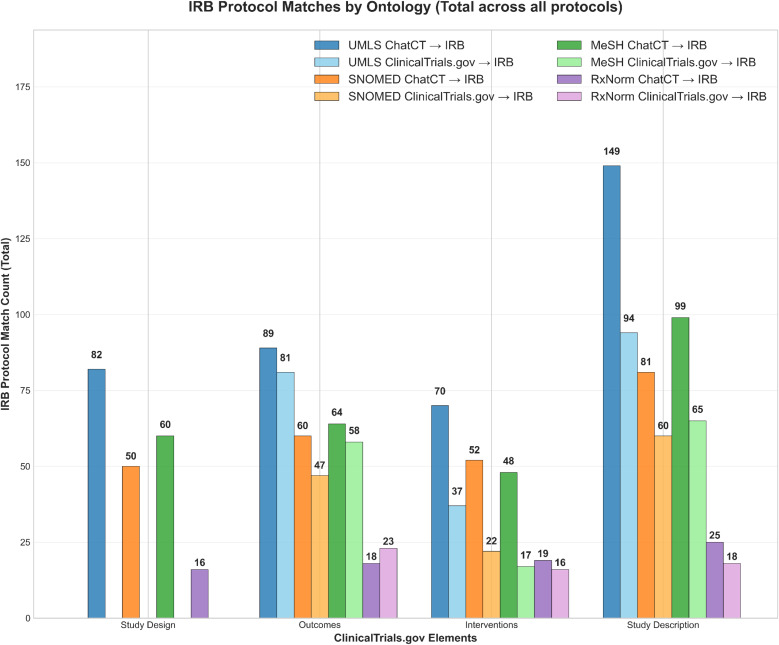
Total count of medical concepts extracted from ClinicalTrials.gov entries and ChatCT outputs that match concepts within the IRB protocol, categorized by four ClinicalTrials.gov elements and four ontologies. IRB = Institutional Review Board.

Figure 4 summarizes protocol-matched concept counts for ChatCT and for the corresponding ClinicalTrials.gov reference entries. ChatCT consistently identified a greater number of protocol-matched concepts across all registry elements and ontologies compared with the corresponding ClinicalTrials.gov entries, particularly for *Study Design* and *Study Description*, where reference entries often contained few or no ontology-matched concepts. Table 2 reports concept-level precision, recall, and F1 scores for both ChatCT-generated outputs and ClinicalTrials.gov reference entries relative to protocol source chunks-derived concepts. Across all elements and ontologies, ChatCT demonstrated high precision, with an overall average precision of 96.7%, indicating that nearly all concepts generated by ChatCT were grounded in the protocol source material. Precision was consistently high across UMLS, SNOMED CT, MeSH, and RxNorm vocabularies, often approaching or reaching 100%. In contrast, ClinicalTrials.gov reference entries showed substantially lower overall precision (average 30.9%), reflecting greater variability in how protocol concepts were represented in human-authored registry records.

**Table 2.** Precision, recall, and F1 scores by ontology and element

Element	Ontology	ChatCT→IRB precision (%)	ChatCT→IRB recall (%)	ChatCT→IRB F1 (%)	ClinicalTrials.gov→IRB precision (%)	ClinicalTrials.gov→IRB recall (%)	ClinicalTrials.gov→IRB F1 (%)
**Study design**	UMLS	96.47	3.78	7.27	0	0	0
	SNOMED	100	4.1	7.87	0	0	0
	MeSH	100	4	7.69	0	0	0
	RxNorm	100	2.87	5.57	0	0	0
**Outcomes**	UMLS	89.9	4.1	7.84	71.05	3.73	7.09
	SNOMED	96.77	4.92	9.36	65.28	3.85	7.28
	MeSH	95.52	4.27	8.17	0	0	0
	RxNorm	100	3.23	6.25	0	0	0
**Interventions**	UMLS	90.91	3.22	6.22	74	1.7	3.33
	SNOMED	96.3	4.26	8.16	95.65	1.8	3.54
	MeSH	96	3.2	6.19	0	0	0
	RxNorm	95	3.41	6.57	0	0	0
**Study description**	UMLS	95.51	6.86	12.8	91.26	4.33	8.26
	SNOMED	98.78	6.64	12.44	96.77	4.92	9.36
	MeSH	96.12	6.6	12.35	0	0	0
	RxNorm	100	4.48	8.58	0	0	0
**SUMMARY (average)**	ALL	96.71	4.37	8.33	30.88	1.27	2.43

IRB = Institutional Review Board.

Recall values were low in absolute terms for both ChatCT and reference entries, with ChatCT achieving a higher average recall (4.37%) than ClinicalTrials.gov entries (1.27%). This pattern reflects the fact that IRB protocols contain a large number of biomedical concepts, many of which are not intended to appear in structured registry fields. Consequently, recall should be interpreted as protocol concept coverage rather than completeness of registry reporting. Despite low recall values, the combination of high precision and comparatively higher recall resulted in higher F1 scores for ChatCT across all registry elements, with an overall average F1 of 8.33%, compared with 2.43% for ClinicalTrials.gov reference entries.

Taken together, these results demonstrate that ChatCT-generated registry drafts consistently remained grounded in protocol source material while capturing a broader and more consistent set of protocol-supported biomedical concepts than existing ClinicalTrials.gov entries.

## Limitations

This study has several limitations. 1. System performance may be affected by increasing protocol length and complexity, particularly when relevant information is distributed across distant sections of the document. Although ChatCT processes full protocols by chunking and embedding the entire text, retrieval is performed over a fixed number of top-ranked chunks per query. As protocol complexity increases, this retrieval strategy may introduce challenges related to context fragmentation and coverage, potentially affecting consistency across generated elements. 2. The evaluation only focused on four registry elements; other required components, such as eligibility criteria and results data, were not assessed in this pilot. 3. ClinicalTrials.gov entries were used as the reference standard despite known variability in human-authored records, which may have penalized AI-generated content that was correct relative to the protocol. 4. Context-dependent distinctions (e.g., disease category vs. specific disease state) were not addressed during manual review of flagged concepts. Finally, the use of a proprietary language model raises considerations related to cost, explainability, data governance, and scalability.

## Discussion

ChatCT generated registry drafts that were semantically faithful to the corresponding human-authored ClinicalTrials.gov entries and largely compliant with structured PRS reporting requirements, while remaining grounded in protocol source text. By capturing protocol-specified details that may be omitted during manual entry, ChatCT demonstrated potential to improve consistency and completeness of registry records. A system such as ChatCT could support protocol-to-registry transcription by generating drafts for ClinicalTrials.gov entries that preserve protocol content and meet most structural requirements, allowing administrative teams to focus on verification. Across the evaluated trials, ChatCT frequently captured protocol-specific details that were not explicitly included in the corresponding registry records, highlighting its potential to improve consistency between protocol documents and public entries. The RAG approach – grounding outputs in IRB-approved protocol text – was central to maintaining high factual accuracy, which is essential for regulatory reporting contexts.

In a small, preliminary use of ChatCT with registry administrators at our institution, we generated drafts for all four registry elements evaluated in this study across three trials. These ChatCT-generated elements were submitted through the PRS system, and for all three trials the drafts were accepted during the initial quality-control review cycle without requests for revision. This observation is reported to illustrate feasibility rather than to demonstrate quantified efficiency gains, as the pilot was not designed to formally evaluate submission timelines or review burden.

Future studies could deploy the tool prospectively during PRS trial registration to systematically measure outcomes such as time to registration completion, number of PRS quality-control review cycles required, and user experience.

## Conclusion

In this study, we developed and evaluated ChatCT, a retrieval-augmented GPT-4–based system for drafting ClinicalTrials.gov registry entries from IRB-approved protocols. This work illustrates how protocol-grounded, retrieval-augmented language models can support clinical trial reporting workflows and enhance the quality and transparency of publicly available trial information.

## Supporting information

10.1017/cts.2026.10735.sm001 Baluguri and Anderson supplementary material Baluguri and Anderson supplementary material

## Supplementary material

The supplementary material for this article can be found at https://doi.org/10.1017/cts.2026.10735.

## References

[1] Chaturvedi N , Mehrotra B , Kumari S , Gupta S , Subramanya HS , Saberwal G. Some data quality issues at ClinicalTrials.gov. Trials. 2019;20:378. doi: 10.1186/s13063-019-3408-2. 31234923 PMC6591874

[2] FDA. Commissioner of the food and drug administration amendments act (FDAAA) of 2007, 2025. (https://www.fda.gov/regulatory-information/selected-amendments-fdc-act/food-and-drug-administration-amendments-act-fdaaa-2007) Accessed October 10, 2025.

[3] National Institutes of Health (NIH). NOT-OD-16-149: NIH policy on the dissemination of NIH-Funded Clinical Trial Information, (https://grants.nih.gov/grants/guide/notice-files/NOT-OD-16-149.html) Accessed October 10, 2025.

[4] Office USGA. National Institutes of Health: better data will improve understanding of federal contributions to drug development | U.S. GAO, (https://www.gao.gov/products/gao-23-105656) Accessed September 8, 2025.

[5] Anderson ML , Chiswell K , Peterson ED , Tasneem A , Topping J , Califf RM. Compliance with results reporting at ClinicalTrials.gov. N Engl J Med. 2015;372:1031–1039. doi: 10.1056/NEJMsa1409364. 25760355 PMC4508873

[6] ClinicalTrials.gov. Protocol registration data element definitions for interventional and observational studies, (https://clinicaltrials.gov/policy/protocol-definitions) Accessed October 10, 2025.

[7] Protocol registration data element definitions for interventional studies, (https://www.nejm.org/doi/full/10.1056/NEJMsr1611785) Accessed October 10, 2025.

[8] ClinicalTrials.gov. Results data element definitions for interventional and observational studies, (https://clinicaltrials.gov/policy/results-definitions) Accessed October 10, 2025.

[9] Zarin DA , Tse T , Williams RJ , Rajakannan T. The status of trial registration eleven years after the ICMJE policy. N Engl J Med. 2017;376:383. doi: 10.1056/NEJMsr1601330. 28121511 PMC5813248

[10] Miron L , Gonçalves RS , Musen MA. Obstacles to the reuse of study metadata in ClinicalTrials. Gov. Scientific Data. 2020;7:443. doi: 10.1038/s41597-020-00780-z. 33339830 PMC7749162

[11] Zhang F , Zhu Y , Zhao S , et al. Discordant information on blinding in trial registries and published research: a systematic review. JAMA Netw Open. 2024;7:e2452274. doi: 10.1001/jamanetworkopen.2024.52274. 39724369 PMC11672156

[12] Amugongo LM , Mascheroni P , Brooks S , Doering S , Seidel J. Retrieval augmented generation for large language models in healthcare: a systematic review. PLOS Digit Health. 2025;4:e0000877. doi: 10.1371/journal.pdig.0000877. 40498738 PMC12157099

[13] npj Digital Medicine. Retrieval augmented generation for 10 large language models and its generalizability in assessing medical fitness, (https://www.nature.com/articles/s41746-025-01519-z) Accessed September 8, 2025. 10.1038/s41746-025-01519-z PMC11971376 40185842

[14] Murcia VM , Aggarwal V , Pesaladinne N , et al. Automating clinical trial matches via natural language processing of synthetic electronic health records and clinical trial eligibility criteria. AMIA Jt Summits Transl Sci Proc AMIA Jt Summits Transl Sci. 2024;2024:125–134. PMC11141802 38827083

[15] Liu L , Blake V , Barman M , et al. Using natural language processing to extract information from clinical text in electronic medical records for populating clinical registries: a systematic review. J Am Med Inform Assn. 2025;33:484–499. doi: 10.1093/jamia/ocaf176. PMC12844598 41093296

[16] Kantor K , Morzy M. Machine learning and natural language processing in clinical trial eligibility criteria parsing: a scoping review. Drug Discov Today. 2024;29:104139. doi: 10.1016/j.drudis.2024.104139. 39154773

[17] Lin A , Wang Z , Jiang A , et al. Large language models in clinical trials: applications, technical advances, and future directions. BMC Med. 2025;23:563. doi: 10.1186/s12916-025-04348-9. 41088200 PMC12522288

[18] U.S. National Library of Medicine. Protocol Registration and Results System (PRS) help [Internet]. Bethesda (MD): ClinicalTrials.gov (https://clinicaltrials.gov/submit-studies/prs-help) Accessed January 12, 2026.

[19] Yang X , He X , Zhang H , Ma Y , Bian J , Wu Y. Measurement of semantic textual similarity in clinical texts: comparison of transformer-based models. JMIR Med Inform. 2020;8:e19735. doi: 10.2196/19735. 33226350 PMC7721552

[20] Zhang T , Kishore V , Wu F , Weinberger KQ , Artzi Y. BERTScore: evaluating text generation with BERT. ArXiv. 2019;abs/1904.09675.

[21] Neumann M , King D , Beltagy I , Ammar W. ScispaCy: fast and robust models for biomedical natural language processing. Proc ACL. 2019.

[22] Zhou W. Biomedical nested NER with large language model and UMLS heuristics. arXiv. Preprint, (10.48550/arXiv.2407.05480) Accessed July 7, 2024.

[23] Bodenreider O , Cornet R , Vreeman DJ. Recent developments in clinical terminologies — SNOMED CT, LOINC, and rxNorm. Yearb Med Inform. 2018;27:129–139. doi: 10.1055/s-0038-1667077. 30157516 PMC6115234

