# Supplementary material for: Retrieval-enhanced drafting of ClinicalTrials.gov data elements from clinical protocols
Source: J Clin Transl Sci. 2026 Mar 31;10(1):e76. doi: 10.1017/cts.2026.10735 (PMC13107080; doi:10.1017/cts.2026.10735)
Supplement: Baluguri and Anderson supplementary material [file S2059866126107353sup001.docx]

**Appendixes**

**Appendix 1**

**Table 1B.** Full Scoring Rubric Table

| **PRS Element** | **PRS Subfield** | **Error Category** | **Penalty** | **PRS Significance** |
| --- | --- | --- | --- | --- |
| Study Design | Study Type | Missing | −0.30 | Required PRS field; omission blocks submission. |
|  | Interventional Study Model | Missing | −0.30 | Mandatory trial classification field. |
|  | Masking | Missing | −0.30 | Required masking roles must be labeled. |
|  | Masking | Format Issue | −0.10 | Narrative 'double-blind' insufficient for PRS. |
|  | Study Phase | Missing | −0.30 | Strict PRS vocabulary requirement. |
|  | Primary Purpose | Missing | −0.30 | Required intent category. |
| Arms & Interventions | Arm Title | Missing | −0.30 | Each arm must be labeled separately. |
|  | Arm Title | Format Issue | −0.10 | Merged arms violate structure. |
|  | Arm Type | Missing | −0.30 | Required to define the role of each arm. |
|  | Intervention Type | Missing | −0.30 | Required FDAAA/PRS intervention classification. |
|  | Intervention Name | Missing | −0.30 | Required non-proprietary naming. |
|  | Intervention Description | Missing | −0.30 | PRS requires descriptive clarity. |
|  | Intervention Description | Length Violation | −0.30 | Exceeds PRS character limit. |
|  | Arm Description | Format Issue | −0.10 | Description must match arm structure. |
|  | Arm Description | Length Violation | −0.30 | Exceeds PRS character limit. |
| Outcome Measures | Outcome Title | Missing Component | −0.10 | Required component of structured Outcome. |
|  | Outcome Description | Missing Component | −0.10 | Metric description required. |
|  | Time Frame | Vague Format | −0.20 | Time frame must be specific. |
|  | Time Frame | Missing Component | −0.10 | Missing required component. |
|  | Primary Outcome Measure | Missing Block | −0.30 | Primary Outcome required for all trials. |
| Study Description | Brief Summary | Missing | −0.30 | Required public-facing summary. |
|  | Detailed Description | Missing | −0.30 | Required technical narrative. |
|  | Detailed Description | Format Issue | −0.10 | Must be separate from the Brief Summary. |

**Appendix 2**

**Table 1.** Distribution of ChatCT runs per protocol–element pair under the frozen configuration

| **Registry Element** | **Total Pairs** | **1 Run (n)** | **>1 Runs (n)** | **Median IQR** | **Min** | **Max** |
| --- | --- | --- | --- | --- | --- | --- |
| Study Design | 29 | 22 | 7 | 0 | 1 | 6 |
| Arms/Interventions | 29 | 20 | 9 | 1 | 1 | 5 |
| Outcome Measures | 29 | 20 | 9 | 1 | 1 | 7 |
| Study Description | 29 | 8 | 21 | 2 | 1 | 6 |

**1 Run =** Number of protocol–element pairs generated exactly once under the frozen configuration.
**>1 Runs =** Number of protocol–element pairs generated more than once under the frozen configuration
